# The potent, indirect adenosine monophosphate-activated protein kinase activator R419 attenuates mitogen-activated protein kinase signaling, inhibits nociceptor excitability, and reduces pain hypersensitivity in mice

**DOI:** 10.1097/PR9.0000000000000562

**Published:** 2016-08-09

**Authors:** Galo L. Mejia, Marina N. Asiedu, Yasumichi Hitoshi, Gregory Dussor, Theodore J. Price

**Affiliations:** a School of Behavioral and Brain Sciences, The University of Texas at Dallas, Richardson, TX, USA; b Rigel Pharmaceuticals Inc, South San Francisco, CA, USA

**Keywords:** AMPK, Nerve growth factor, Complex I inhibitor, Incision pain

## Abstract

There is a great need for new therapeutics for the treatment of pain. A possible avenue to development of such therapeutics is to interfere with signaling pathways engaged in peripheral nociceptors that cause these neurons to become hyperexcitable. There is strong evidence that mitogen-activated protein kinases and phosphoinositide 3-kinase (PI3K)/mechanistic target of rapamycin signaling pathways are key modulators of nociceptor excitability in vitro and in vivo. Activation of adenosine monophosphate-activated protein kinase (AMPK) can inhibit signaling in both of these pathways, and AMPK activators have been shown to inhibit nociceptor excitability and pain hypersensitivity in rodents. R419 is one of, if not the most potent AMPK activator described to date. We tested whether R419 activates AMPK in dorsal root ganglion (DRG) neurons and if this leads to decreased pain hypersensitivity in mice. We find that R419 activates AMPK in DRG neurons resulting in decreased mitogen-activated protein kinase signaling, decreased nascent protein synthesis, and enhanced P body formation. R419 attenuates nerve growth factor (NGF)-induced changes in excitability in DRG neurons and blocks NGF-induced mechanical pain amplification in vivo. Moreover, locally applied R419 attenuates pain hypersensitivity in a model of postsurgical pain and blocks the development of hyperalgesic priming in response to both NGF and incision. We conclude that R419 is a promising lead candidate compound for the development of potent and specific AMPK activation to inhibit pain hypersensitivity as a result of injury.

## Abstract 2

Supplemental Digital Content is Available in the Text.

## 1. Introduction

Endogenous pro-nociceptive factors are released in response to injury and act via their cognate receptors expressed by nociceptive sensory neurons in the dorsal root ganglion (DRG) to alter the excitability of these neurons.^5^ This nociceptive amplification mechanism can result in primary hyperalgesia, ongoing or spontaneous pain, and may be an early signaling event that can lead to the development of a chronic pain state.^47,48^ Pro-nociceptive factors that act via tyrosine receptor kinases and/or G-protein-coupled receptors activate signaling in the mitogen-activated protein kinase (MAPK) and phosphoinositide 3-kinase (PI3K)/mechanistic target of rapamycin (mTOR) pathways. These signaling pathways can lead to the phosphorylation of voltage-gated channels^19,54,59^ and an increase in protein synthesis via convergent signaling to the eukaryotic initiation factor (eIF) proteins.^24,33,34,44,45^ Both of these events are linked to increased excitability in nociceptors but they can be difficult to attenuate by using kinase or enzyme inhibitors that target single signaling factor because feedback signaling is a frequent consequence of PI3K/mTOR- or MAPK-specific antagonists.^7,35,41^

A possible solution to this problem is to target the adenosine monophosphate-activated protein kinase (AMPK) that attenuates PI3K/mTOR and/or MAPK signaling and short-circuits feedback signaling.^35,42,43^ Previous studies in rodents have shown that AMPK activators attenuate inflammatory pain,^2,50^ incision-evoked pain,^56^ trauma-induced neuropathic pain,^34,35^ diabetic neuropathic pain,^28^ bone cancer pain,^53^ and chemotherapy-induced peripheral neuropathic pain.^30^ Direct application of AMPK activators to nociceptive neurons decreases their excitability and inhibits signaling in PI3K/mTOR and MAPK signaling pathways.^34,35,56^ Adenosine monophosphate-activated protein kinase can be activated indirectly by inhibiting mitochondrial complex I activity that raises intracellular AMP/ATP ratios, indirectly through changes in intracellular calcium, or directly through positive allosteric modulators that interact with sites on the heterotrimeric enzyme complex.^15,16^ The antidiabetic drug metformin activates AMPK at least in part by inhibition of mitochondrial complex I,^52^ but metformin does this only at high μM/mM concentrations and requires an organic cation transporter to enter cells.^9^ Despite these limitations, metformin is effective in many of the preclinical models listed above, and retrospective studies in humans^55^ suggest a beneficial effect on pain.

As a first step toward the development of more potent and specific complex I inhibitors that activate AMPK for the treatment of pain, we have examined the effect of R419 on nociceptive neurons in vitro and in vivo. R419 activates AMPK at nM concentrations,^20^ making it one of the most potent AMPK activators yet described. We find that R419 activates AMPK in DRG neurons, leading to inhibition of MAPK signaling and a robust decrease in cellular excitability. Moreover, R419 blocks nerve growth factor (NGF)- and incision-evoked mechanical hypersensitivity and hyperalgesic priming via local injection. Our findings support the further development of regionally applied AMPK activators for the beneficial modification of injury-induced nociceptive plasticity.

## 2. Methods

### 2.1. Experimental animals

All procedures that involved use of animals were approved by the Institutional Animal Care and Use Committee of The University of Texas at Dallas and were in accordance with International Association for the Study of Pain guidelines. All behavioral studies were conducted using male Swiss Webster (Taconic Laboratories) mice weighing between 20 and 25 g. Mice were used in behavioral experiments starting 1 week after arrival at the animal facility at University of Texas at Dallas. Animals were housed with a 12-hour light/dark cycle and had food and water available ad libitum.

### 2.2. Behavioral testing and drug administration

Mice were placed in acrylic boxes with wire mesh floors and allowed to habituate for 1 hour. After pretreatment mechanical thresholds were recorded, animals received intraplantar injections in a volume of 25 μL sterile saline. Other mice had plantar incision surgery, using a model first described by Pogatzki and Raja^40^ and modified by Banik et al.^4^ We made incisions in the skin and underlying muscle prior to suturing the wound as described by Banik et al.^4^ Animals that had plantar incision were briefly anesthetized with isoflurane during the procedure. Calibrated von Frey filaments (Stoelting) were used for mechanical stimulation of the plantar surface of the left hind paw, and withdrawal thresholds were calculated using the up-down method (Chaplan et al.^8^). R419 (synthesized at Rigel, San Francisco, CA^20^) was made as a DMSO stock solution and then diluted into dosing vehicles. Dosing was carried out in a volume of 25 μL sterile saline for intraplantar injections or in 0.5% hydroxypropyl methylcellulose plus 0.1% polysorbate-80 (both from Sigma, St. Louis, MO) in ultrapure H_2_O in a total volume of 200 μL for oral gavage. The experimenters measuring mechanical withdrawal thresholds were always blinded to the experimental conditions. Mice were randomized to groups by a blinded experimenter, and mice of individual groups were never housed together (eg, home cages were always mixed between experimental groups).

### 2.3. Tissue culture, Western blotting, cellular imaging, and electrophysiology

Dorsal root ganglia (DRGs) were extracted aseptically from Swiss Webster mice for each Western blot or cellular imaging experiment and placed in Hank's Buffered Salt Solution (Invitrogen, Waltham, MA) on ice. Following DRG neuron dissociation, neurons were cultured for 7 days on 12-well plates coated with poly-d-lysine (Falcon, Franklin Lakes, NJ) for Western blot experiments or on 12-mm glass coverslips (No. 1 thickness; Chemglass, Vineland, NJ) in a 24-well tissue culture plate (Falcon) at 37°C with 95% air and 5% CO_2_. Harvested tissues were homogenized in lysis buffer, protease inhibitor cocktail (P8340; Sigma), and phosphatase inhibitor cocktail 2/3 (P5726, P0044; Sigma) and prepared for standard Western blotting with polyvinylidene difluoride (PVDF) membranes (Millipore, Billerica, MA). Densitometric analyses were performed on immunoblots with ImageJ software (NIH, Bethesda, MD). Antibodies used are shown in Table 1.

**Table 1:**
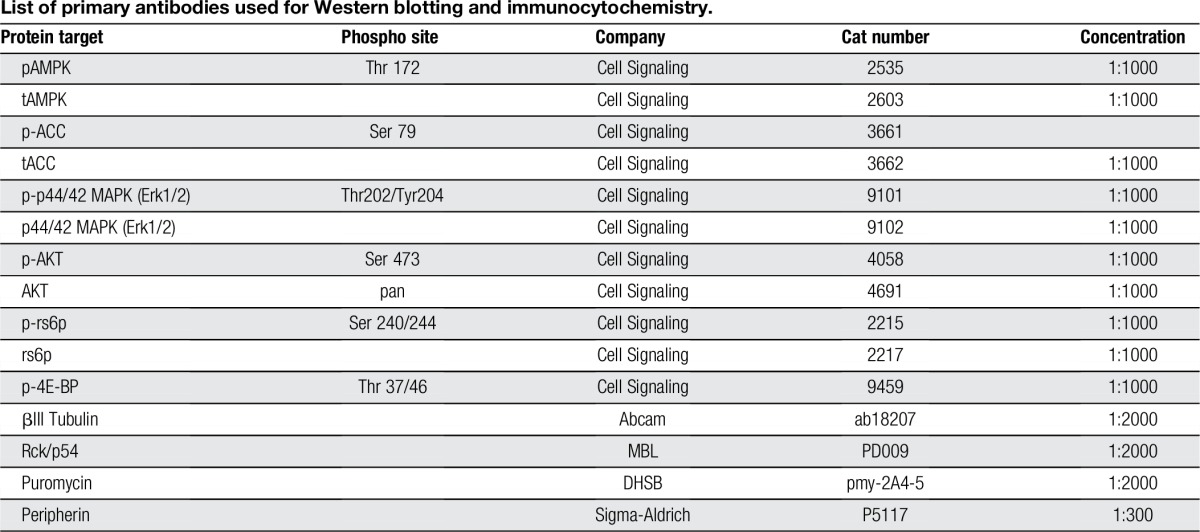
List of primary antibodies used for Western blotting and immunocytochemistry.

For immunocytochemistry, treatments were performed to assess acetyl-CoA carboxylase (ACC) phosphorylation, P body formation,^1^ and protein puromycelation. For the puromycelation, surface sensing of translation (SUnSET) assay,^51^ puromycin (1 μM; Sigma) was applied during the last 15 minutes of the 1-hour incubation with drug. Immediately following the puromycin incubation, cells were washed in chilled Hank's Buffered Salt Solution containing 0.00036% digitonin (Sigma) for 2 minutes prior to fixation for the removal of background puromycin. Images were taken on an Olympus Fluoview FV1200 laser scanning confocal microscope and analyzed using the colocalization tool within Olympus FV software.

Whole cell patch-clamp experiments were performed on isolated DRG neurons in vitro using a MultiClamp 700B (Axon Instruments, Sunnyvale, CA) patch-clamp amplifier and PClamp 9 acquisition software (Axon Instruments). Recordings were sampled at 20 kHz and filtered at 1 kHz. Series resistance was typically <7 MΩ and was compensated 60% to 80%. Data were analyzed using Clampfit 10 (Molecular Devices, Sunnyvale, CA) and Origin 8 (OriginLab, Northampton, MA). Action potentials were elicited by injecting slow ramp currents from 0.1 to 0.7 nA with Δ = 0.2 nA over 1 second to mimic slow depolarization.

Detailed methods are available in Supplemental Methods (available online at http://links.lww.com/PR9/A0).

### 2.4. Data analysis and statistics

All data are presented as mean ± SEM. Data were analyzed with GraphPad (San Diego, CA) Prism version 6 for PC or Mac. Statistical tests used are described in figure legends. The a priori level of significance was set at 95%.

## 3. Results

### 3.1. R419 activates adenosine monophosphate-activated protein kinase in dorsal root ganglion neurons to inhibit mitogen-activated protein kinase signaling and attenuate nascent protein synthesis

**Figure 1.**
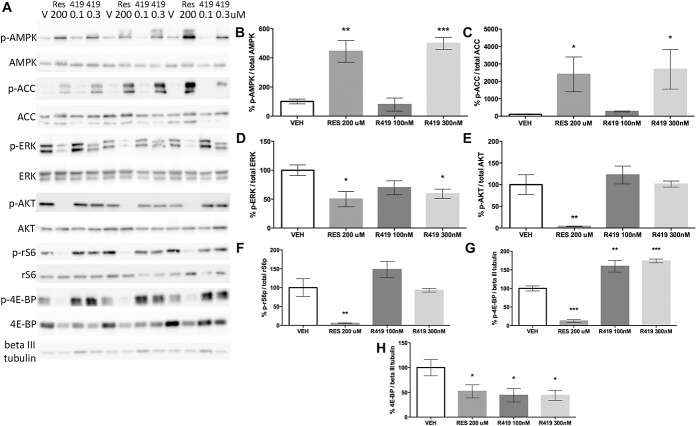
R419 potently activates AMPK in mouse DRG neurons, leading to inhibition of ERK signaling. (A) Western blots are shown for triplicate samples of DRG neurons in culture for 7 days from male Swiss Webster mice exposed to vehicle (V), resveratrol (RES, 200 μM), R419 (419, 0.1, and 0.3 μM) for 1 hour. Twenty micrograms of protein was loaded per lane and transferred to membranes that were blotted for phospho and total AMPK (B), ACC (C), ERK (D), AKT (E), rS6 (F), and 4E-BP (G and H) as well as βIII tubulin as a loading control. Data are plotted as the percentage change for the phosphorylated form of the protein divided by the total protein quantification standardized to the vehicle (VEH) treatment. 4E-BP and p-4E-BP were compared with the βIII tubulin loading control. Ordinary one-way analysis of variance was used to assess differences between the vehicle and treatment groups with Fisher least significant difference post hoc test. *P < 0.05, **P < 0.01, ***P < 0.001. N = 3 per group with the exception of panel D where the N = 6. AMPK, adenosine monophosphate-activated protein kinase.

We first used Western blotting on samples generated from mouse DRG neurons in culture to assess whether R419 activates AMPK and inhibits downstream signaling pathways in these primary cultures (Fig. 1A). We used 200 μM resveratrol as a positive control because we have previously shown that this compound activates AMPK in these neurons to inhibit MAPK and mTOR signaling.^56^ One-hour treatment with both 100 μM resveratrol and 300 nM R419 led to increased phosphorylation of AMPK at threonine 172 (Fig. 1B). Consistent with this finding, 200 μM resveratrol and 300 nM R419 increased phosphorylation of ACC at serine 79, a phosphorylation site that is targeted specifically by activated AMPK (Fig. 1C). In DRG cultures, we observed bands for ACC at 280 and 265 kD, indicating that both the lipogenic and oxidative ACC isoforms are expressed in DRG cultures.

We then assessed whether R419 inhibits MAPK or PI3K/mTOR signaling in DRG neurons. Again, we used resveratrol as a positive control for these experiments because we have previously shown that this compound inhibits both of these signaling pathways.^56^ Both 200 μM resveratrol and 300 nM R419 led to a ∼50% reduction in ERK1/2 phosphorylation at threonine 202/185 and tyrosine 204/187, respectively, in DRG neurons (Fig. 1D). In contrast, resveratrol inhibited AKT phosphorylation at serine 473 (Fig. 1E), ribosomal S6 protein (rS6) phosphorylation at serine 240/244 (Fig 1F), and 4E-BP phosphorylation at threonine 37/46 (Fig. 1G), whereas R419 had no effect on these phosphorylated proteins. R419 increased 4E-BP phosphorylation relative to βIII tubulin levels (Fig. 1G), and both resveratrol and R419 decreased overall 4E-BP protein levels compared with βIII tubulin loading control (Fig. 1H). These findings indicate that R419 activates AMPK and inhibits MAPK signaling but fails to attenuate PI3K/mTOR signaling in DRG neurons.

**Figure 2.**
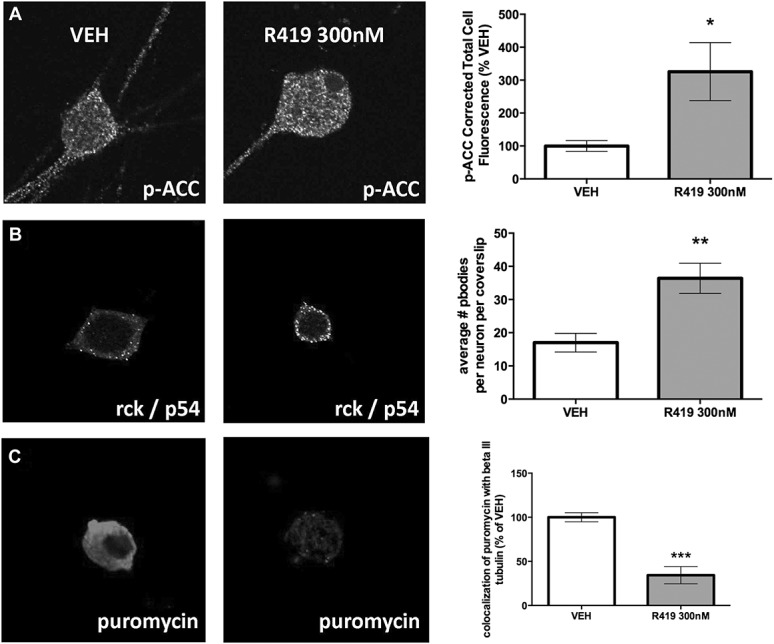
R419 increases AMPK signaling in DRG neurons leading to increased P body formation and inhibition of nascent protein synthesis. (A) DRG neurons in culture taken from male Swiss Webster mice were treated with R419 for 1 hour and then assayed for p-ACC and assessed by confocal microscopy for changes in ACC phosphorylation in neurons labeled with βIII tubulin, N = 9 (VEH) or 10 (R419) coverslips. (B) DRG neurons in culture were treated with R419 for 1 hour and then assayed for P body formation with the P body marker rck/p54 and assessed by confocal microscopy for changes in puncta indicative of P body formation, N = 11 (VEH) or 10 (R419) coverslips. (C) SUnSET assay was used to examine nascent protein synthesis in DRG neurons treated with R419 for 1 hour. Puromycin incorporation into proteins was measured with a puromycin antibody and assessed in βIII tubulin–positive DRG neurons, N = 6 (VEH) or 8 (R419) coverslips. Differences between groups were assessed by 2-tailed Student *t* test. *P < 0.05, **P < 0.01, ***P < 0.001. ACC, acetyl-CoA carboxylase; AMPK, adenosine monophosphate-activated protein kinase; DRG, dorsal root ganglion.

Next, we sought to examine cellular effects in DRG neurons with AMPK activation through R419. Because DRG cultures contain neurons, fibroblasts, Schwann cells, satellite glial cells, and other cell types, it is important to demonstrate that R419 is capable of stimulating AMPK-mediated actions in DRG neurons. We first looked at ACC phosphorylation on serine 79 using a phospho-specific antibody characterized for immunocytochemistry. Using corrected total cellular fluorescence on neurons labeled with βIII tubulin, we observed a significant increase in p-ACC with R419 (300 nM) treatment for 1 hour (Fig. 2A). We have previously shown that AMPK activation causes an increase in P body formation in DRG neurons.^36^ P bodies are sites of RNA processing in cells, and P body formation is associated with decreased mRNA translation.^1^ Therefore, we also used the SUnSET assay^51^ to label nascently synthesized proteins with puromycin (1 μM treatment for 15 minutes). Consistent with previous studies, we observed an increase in P body formation in DRG neurons (Fig. 2B) and a decrease in puromycelation (Fig. 2C) upon treatment with R419 (300 nM). To assess specificity of R419, we screened 10 μM R419 against a broad variety of receptors and enzymes. R419 only displayed greater than 50% activity at the dopamine reuptake transporter (supplementary table 1, available online at http://links.lww.com/PR9/A0), suggesting that R419 has excellent specificity at nM concentrations. From these findings, we conclude that R419 activates AMPK in DRG neurons and that this leads to a decrease in ERK activity, enhanced P body formation, and decreased mRNA translation.

### 3.2. R419 blocks nerve growth factor–mediated mechanical hypersensitivity, hyperalgesic priming, and cellular excitability

**Figure 3.**
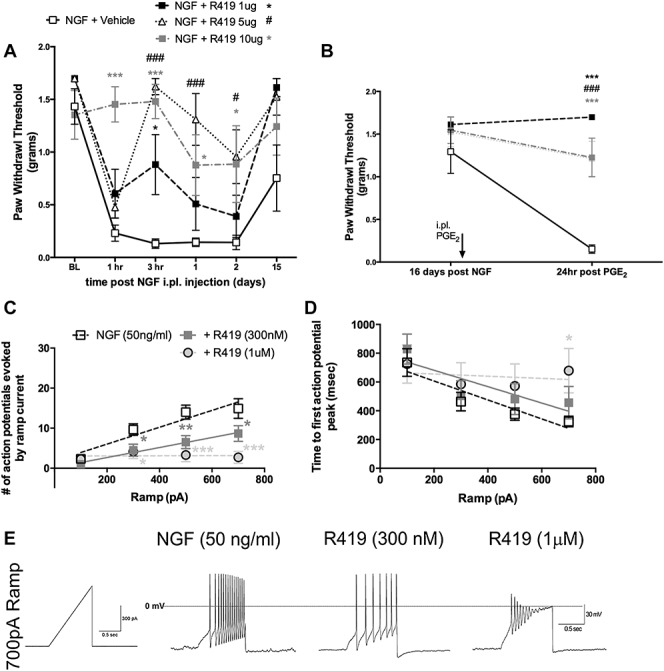
R419 inhibits NGF-evoked mechanical hypersensitivity and NGF-mediated DRG neuron excitability. (A) Male Swiss Webster mice were treated with NGF (50 ng) ± R419 at the indicated doses and assessed for mechanical sensitivity by von Frey hair stimulation at the indicated time points, N = 6 per group. (B) Mice were subsequently assessed for hyperalgesic priming with injection of PGE_2_ 16 days after NGF ± R419 treatment, N = 6 mice per group. (C) Small-diameter DRG neurons were exposed to NGF overnight and then exposed to vehicle or the indicated concentrations of R419 for 1 hour. Ramp currents were used to evoke spiking in recorded neurons. The number of action potentials evoked by the ramp current is shown in (C), and the time to first action potential peak evoked by the ramp current is shown in (D). An example trace for the 3 conditions in (C) and (D) for the 700 pA ramp is shown in (E). NGF alone group, N = 12; NGF + R419 300 nM, N = 6; R419 1 μM, N = 7. Behavioral data were analyzed by 2-way ANOVA with Bonferroni post hoc test. Electrophysiology data were analyzed by 2-way ANOVA with Fisher LSD post hoc test. *P < 0.05, **P < 0.01, ***P < 0.001. # *P* < 0.05, ### *P* < 0.001 for NGF versus NGF + R419 5 ug group. ANOVA, analysis of variance; DRG, dorsal root ganglion; NGF, nerve growth factor.

Nerve growth factor sensitizes nociceptors in rodents and humans.^32^ Nerve growth factor signals through MAPK and PI3K/mTOR pathways to evoke changes in nociceptor excitability and induce mechanical hypersensitivity in vivo.^33,45^ We tested whether R419 could block NGF-induced changes in behavior in vivo or in cellular excitability in vitro. Mice received intraplantar injections of NGF with or without R419, and mechanical hypersensitivity was assessed by the von Frey test. R419 dose-dependently inhibited mechanical hypersensitivity with a peak effect at 10 μg dose (Fig. 3A). We also assessed the presence of hyperalgesic priming 16 days after NGF treatment, a time point where animals had completely returned to baseline mechanical thresholds, by giving an intraplantar injection of PGE_2_. All doses of R419 effectively blocked the development of hyperalgesic priming in response to NGF injection (Fig. 3B).

Next, we cultured DRG neurons from mice and exposed them to NGF (50 ng/mL) overnight. We treated DRG neurons with vehicle or 2 concentrations of R419 (300 and 1000 nM) for 1 hour prior to performing current clamp recordings on small-diameter DRG neurons. We assessed ramp current-evoked spiking and measured the number of evoked action potentials and the time to first action potential peak with each ramp current. R419 inhibited the number of action potentials evoked by ramp currents in a concentration-dependent fashion (Fig. 3C). R419 treatment did not change the time to first action potential peak except at the highest dose with the strongest ramp current injection (Fig. 3D). An example trace for the 700 pA ramp current for vehicle, 300 nM, and 1000 nM R419 is shown in Figure 3E. These experiments demonstrate that R419 completely blocks NGF-evoked mechanical hypersensitivity and hyperalgesic priming in vivo and strongly attenuates DRG excitability in neurons exposed to NGF.

### 3.3. R419 inhibits incision-evoked pain

**Figure 4.**
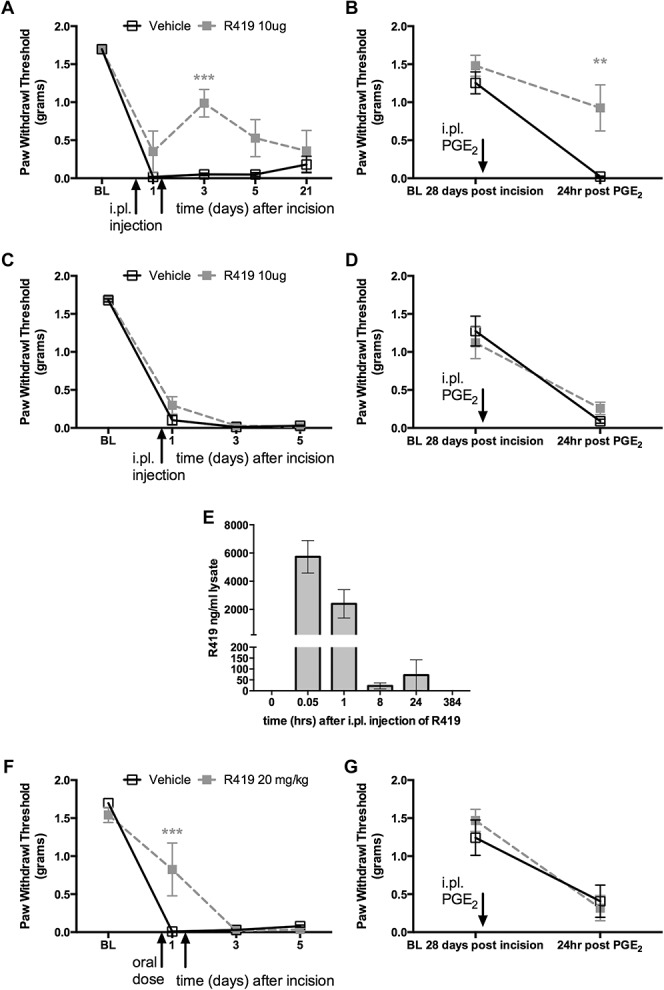
R419 inhibits incision-evoked mechanical hypersensitivity but has a short tissue half-life. (A) Male Swiss Webster mice underwent plantar incision surgery and received intraplantar (i.pl.) injections of R419 (10 μg) or vehicle at the time of incision and 24 hours after incision. Mechanical thresholds were assessed at the indicated time points and hyperalgesic priming (B) was measured by PGE_2_ response at 28 days after incision, when animals had completely recovered from the initial hypersensitivity, N = 6 per group. (C and D) Similar experiments were conducted but with a single i.pl. injection at the time of incision only, N = 6 per group. (E) R419 levels in the hind paw skin samples were measured by HPLC at the indicated time points, suggesting a t_1/2_ of approximately 1 hour for R419, N = 4 per group. (F and G) Efficacy of R419 given by oral gavage (20 mg/kg) was tested in the plantar incision and hyperalgesic priming model with dosing at 1 hour prior to incision and again 24 hours after surgery, N = 6 per group. **P < 0.01, ***P < 0.001.

Nerve growth factor is produced at the site of surgical incision where it promotes changes in excitability in nociceptors that innervate the incision area.^3,61,63^ The findings above show that R419 blocks NGF-mediated changes in excitability in DRG neurons. We have previously shown that the AMPK activator resveratrol attenuates incision-evoked pain and hyperalgesic priming caused by incision. Based on this logical framework, we examined whether R419 would inhibit incision-evoked pain in mice. We first used a dosing schedule that was effective with resveratrol^56^: local injection at the time of incision and injection at the incision site 24 hours after surgery. With local R419 (10 μg) injection, we observed the inhibition of incision-evoked mechanical hypersensitivity (Fig. 4A) and blockade of hyperalgesic priming when animals were challenged with PGE_2_ 28 days after the incision (Fig. 4B). Based on this strong effect, we also tested whether a single injection of R419 (10 μg) at the time of surgery would be efficacious. In this study, we did not observe any effect on acute mechanical hypersensitivity (Fig. 4C) or on hyperalgesic priming (Fig. 4D). We suspected that the dependence on multiple dosing for efficacy could suggest a relatively short t_1/2_ for R419 at the site of injection. To test this, we injected naïve mice with R419 and took tissue samples at several time points. We observed a sharp peak in R419 levels shortly after injection that was cut to roughly half within 1 hour (Fig. 4E). By 24 hours, tissue concentration had fallen to less than 100 ng/mL, and R419 was absent at 384 hours after injection. We conclude that R419 has a tissue t_1/2_ of approximately 1 hour, providing a possible explanation for why multiple dosing is needed to induce antihyperalgesia following incision.

Previous work has shown that R419 is orally available and activates AMPK-dependent processes in liver, skeletal muscle, and adipose tissue for at least 60 minutes after oral dosing.^20^ We tested whether oral R419 would influence incision-evoked mechanical hypersensitivity when administered 1 hour prior to incision and again 24 hours later. In this study, we saw a transient inhibition of mechanical hypersensitivity (Fig. 4F), but this effect did not carry over to inhibition of hyperalgesic priming when mice were challenged with PGE_2_ 28 days later (Fig. 4G).

## 4. Discussion

The experiments described above show that: (1) R419 is a potent, specific inhibitor of mitochondrial complex I leading to activation of AMPK in DRG neurons, (2) R419-mediated AMPK activation results in inhibition of ERK signaling but does not disrupt PI3K/mTOR signaling, (3) R419 blocks nascent protein synthesis in DRG neurons and increases P body formation, (4) R419 inhibits NGF-evoked mechanical hypersensitivity, hyperalgesic priming, and neuronal hyperexcitability, and (5) multiple, local dosing with R419 blocks postsurgical pain and hyperalgesic priming produced by incision. These findings add to a growing body of evidence, suggesting that AMPK activation can lead to inhibition of pain amplification mechanisms in the peripheral nervous system.^42,43^ R419 may be a clinical candidate for the testing of AMPK activation as a pain therapeutic mechanism in humans.

Previous work assessing AMPK activators for the treatment of pain has focused largely on drugs that are already clinically available (eg, metformin^28,30,34,35,50,53^), on natural products that are generally regarded as safe (eg, resveratrol^49,56^), or on tool compounds that activate AMPK but lack potent actions that are desirable in clinical candidate compounds (eg, AICAR or A769662^34,50^). While all of these compounds have shown efficacy in several preclinical models of persistent inflammatory pain, neuropathic pain or postsurgical pain, A769662 is the most potent of these molecules with an EC_50_ for AMPK activation in the 50 to 200 μM range depending on the cellular assay.^10^ Importantly, R419 is more than 100 times more potent than these other tool compounds with previously reported EC_50_s in the 100 to 300 nM range,^20^ a potency which we have confirmed here on DRG neurons using several different assays.

We have confirmed previous findings in trigeminal neurons from our laboratory^56^ showing here that resveratrol inhibits both MAPK and PI3K/mTOR signaling in DRG neurons. R419, on the other hand, inhibited MAPK signaling but did not influence PI3K/mTOR signaling, at least on the timescale of testing performed in this study. A recent study found that long-term R419 systemic dosing leads to lowered fasting insulin, improved glucose tolerance, and enhanced exercise capacity in an AMPK-dependent fashion. This study assessed AKT phosphorylation in muscle and liver and found no indication of decreased AKT activity with R419 treatment,^31^ consistent with our findings. Adenosine monophosphate-activated protein kinase activation leads to phosphorylation of a variety of downstream targets including ACC, IRS1, B-Raf, and raptor,^17^ and phosphoproteomic methods have recently unveiled new AMPK targets.^18^ While it is currently unclear why certain AMPK activators preferential engage different segments of this signaling pathway, such signaling selectivity may be advantageous in certain pathological states. For instance, in the setting of nociceptor plasticity, while acute inhibition of mTORC1 reduces nociceptor excitability,^13,22,46^ persistent mTORC1 inhibition increases nociceptor excitability through engagement of feedback signaling processes.^35^ Mitogen-activated protein kinase signaling, in particular ERK and p38, is a well-known mechanism for sensitization of nociceptors.^21^ This likely involves 3 major events, direct phosphorylation of ion channels by ERK and p38,^19,54^ regulation of translation via signaling to the eIF4F complex,^33,34^ and regulation of transcription.^21^ We provide evidence that R419 engages 2 of these pathways. First, we show that R419 decreases nascent protein synthesis in DRG neurons using the SUnSET assay. Second, we show that R419 inhibits NGF-induced changes in ramp current-evoked spiking, an effect that has previously been linked to MAPK-mediated phosphorylation of Nav1.7 and/or Nav1.8.^19,54^ Mitogen-activated protein kinase activation in vivo promotes the development of mechanical hypersensitivity caused by a wide variety of algogens, such as NGF.^21,33^ We also find that R419 inhibits NGF-induced mechanical hypersensitivity and hyperalgesic priming. All of these findings are consistent with an AMPK signaling to MAPK-mediated mechanism, suggesting that engagement of this arm of AMPK signaling is sufficient for beneficial effects on pain amplification mechanisms in the periphery.

The mechanism of action of R419 is mitochondrial complex I inhibition, an effect which raises AMP levels in cells causing AMPK activation.^20^ Dysregulation of mitochondrial function has been implicated in a several forms of neuropathic pain, most notably chemotherapy-induced peripheral neuropathic pain.^6,11,12,26^ In this study, it is notable that other complex I inhibitors (such as rotenone) have been shown to be effective against chemotherapy-induced peripheral neuropathic pain in rodents.^14,23,62^ Metformin, which also inhibits complex I to activate AMPK,^39^ was recently shown to prevent the development of chemotherapy-induced peripheral neuropathic pain in mice.^30^ This suggests that a possible endpoint for complex I inhibitors for the attenuation of pain is the activation of AMPK. Metformin has activity at other targets, such as inhibition of AMP deaminase,^38^ inhibition of cyclic AMP signaling,^37^ and inhibition of mitochondrial glycerophosphate dehydrogenase^29^; therefore, it is difficult to reach firm conclusions about mechanisms of action with metformin. It is also clear that clinical concentrations of metformin^58^ may not align with the concentrations of drug that are achieved in preclinical models,^60^ creating further uncertainty about how mechanism of action relates to clinical or preclinical phenotype. We believe that our work with R419 brings clarity to this issue. R419 is a potent and specific complex I inhibitor that robustly activates AMPK at nM concentrations. Our work supports a role for complex I inhibition as a mechanism for AMPK activation in sensory neurons and suggests that previously observed pain-modifying effects of other complex I inhibitors may be attributable to AMPK activation. This is particularly important in light of the recent findings that AMPK activation is required to link mitochondrial fragmentation with mitochondrial fission and mitophagy to eliminate and replace damaged mitochondria.^57^

R419 was more efficacious when given locally than when administered systemically. While we did not perform dosing titers to determine equivalent drug concentrations with local vs oral administration, we did measure drug concentration in the hind paw following local injection. Our findings demonstrate a tissue half-life of ∼1 hour for R419. R419 effectively inhibited NGF-induced mechanical hypersensitivity and hyperalgesic priming with a single dose and required 2 doses for efficacy against incision-evoked mechanical hypersensitivity and hyperalgesic priming. This is likely because incision leads to long-lasting increases in NGF^3,63^ (and other pro-nociceptive factors) at the site of incision, whereas injection of NGF only introduces the factor exogenously for a short period of time. Nevertheless, it is remarkable that even with this short tissue half-life, R419 is capable of producing a long-lasting modification of pain plasticity with limited local dosing. We propose that this creates a promising therapeutic approach for AMPK activators. Local application of drugs is increasingly being used in therapeutics because it allows for spatial control of drug concentrations without risk of systemic toxicities or other side effects (eg, central nervous system effects). A similar complex I inhibitor that activates AMPK was recently shown to produce lactic acidosis with systemic administration in humans.^27^ Our work suggests that local application of AMPK activators at sites of surgery can reduce postsurgical pain and prevent the development of long-lasting nociceptive plasticity that may influence the transition to a chronic pain state^24,45,48^ while avoiding important negative systemic effects in patients.^27^ Insofar, as chronic pain after surgery remains a major clinical problem,^25^ AMPK activators like R419 may be used advantageously via local application in this setting.

We conclude that R419 activates AMPK in sensory neurons leading to inhibition of ERK signaling, decreased nascent protein synthesis, attenuated nociceptor excitability, and locally mediated inhibition of pain plasticity in vivo. Our work shows that very potent, indirect activators of AMPK can modify pain responses in vivo even with relatively brief drug exposure. This creates opportunities for further development of AMPK activators as analgesics with efficacy via a local mechanism of action.

## Conflict of interest statement

The authors have no conflicts of interest to declare.

This work was supported by NIH grants: R01GM102575 (T.J.P. and G.D.), R01NS065926 (T.J.P.) and The University of Texas STARS program research support grant (T.J.P. and G.D.) and by an unrestricted grant from Rigel Pharmaceuticals (T.J.P. and G.D.). Y. Hitoshi is an employee of Rigel Pharmaceuticals.

## Supplementary Material

SUPPLEMENTARY MATERIAL

## Appendix A Supplemental Digital Content

Supplemental Digital Content associated with this article can be found online at http://links.lww.com/PR9/A0.
