# Supplementary material for: The potent, indirect adenosine monophosphate-activated protein kinase activator R419 attenuates mitogen-activated protein kinase signaling, inhibits nociceptor excitability, and reduces pain hypersensitivity in mice
Source: Pain Rep. 2016 Aug 9;1(1):e562. doi: 10.1097/PR9.0000000000000562 (PMC5034875; doi:10.1097/PR9.0000000000000562)
Supplement: SUPPLEMENTARY MATERIAL [file painreports-1-e562-s001.docx]

**Supplemental Materials**

**Supplemental methods**

*Experimental animals:*

All procedures that involved use of animals were approved by the Institutional Animal Care and Use Committee of The University of Texas at Dallas and were in accordance with International Association for the Study of Pain guidelines. All behavioral studies were conducted using male Swiss Webster (Taconic Laboratories) mice weighing between 20 and 25 grams. Mice were used in behavioral experiments starting one week after arrival at the animal facility at University of Texas at Dallas. Animals were housed with a 12 hr light/dark cycle and had food and water available *ad libitum*.

*Behavioral Testing and Drug Administration:*

Mice were placed in acrylic boxes with wire mesh floors and allowed to habituate for 1 hr. After pretreatment mechanical thresholds were recorded, animals received intraplantar injections of NGF (50 ng, Millipore, Billerica, MA) in a volume of 25 μL sterile saline. Other mice had plantar incision surgery, using a model first described by Pogatzki and Raja [40] and modified by Banik et al [4]. We made incisions in the skin and underlying muscle prior to suturing the wound as described by Banik et al [4]. Animals that had plantar incision were briefly anesthetized with isoflurane during the procedure. Calibrated Von Frey filaments (Stoelting) were used for mechanical stimulation of the plantar surface of the left hindpaw and withdrawal thresholds were calculated using the up-down method (Chaplan et al., 1994). For assessment of hyperalgesic priming, animals were baselined for mechanical thresholds after recovery from NGF or incision mechanical hypersensitivity and subsequently injected in the left hindpaw with 100 ng of prostaglandin E_2_ (PGE_2_, Cayman Chemical, Ann Arbor, MI, USA) in 25 μl sterile 0.9% NaCl. Afterward, mechanical withdrawal thresholds were measured at 3 and 24 hr. R419 (synthesized at Rigel, San Francisco, CA, [20]) was made as a DMSO stock solution and then diluted into dosing vehicles. Dosing was done in a volume of 25 μL sterile saline for intraplantar injections or in 0.5% hydroxypropyl methylcellulose plus 0.1% polysorbate-80 (both from Sigma, St. Louis, MO, USA) in ultrapure H_2_O in a total volume of 200 μL for oral gavage.

The experimenters measuring mechanical withdrawal thresholds were always blinded to the experimental conditions. Mice were randomized to groups by a blinded experimenter and mice of individual groups were never housed together (e.g. home cages were always mixed between experimental groups). We noted that the duration of mechanical hypersensitivity in response to NGF or incision was longer in Swiss Webster mice than we have previously observed in ICR mice [56] or than other investigators have observed in several other strains [4]. Swiss Webster outbred mice also display more intense mechanical hypersensitivity than ICR or C57Bl6 mice following spared nerve injury surgery (Price and Mejia, unpublished observations) suggesting a generalized greater pain response in this strain of mice.

*Tissue Culture for Western Blotting and Cellular Imaging:*

Dorsal root ganglia (DRG) were extracted aseptically from 8 4-week old male Swiss Webster mice per cell culture plate for each Western blot or cellular imaging experiment and placed in Hank’s Buffered Salt Solution (HBSS, Invitrogen) on ice. The ganglia were dissociated enzymatically at 37° C; first with collagenase A (1 mg/ml, Roche) for 25 min, then collagenase D (1 mg/ml, Roche) that included papain (30 μg/ml, Roche) for 20 min. Afterwards, a trypsin inhibitor (1 mg/ml, Roche) that contained bovine serum albumin (BSA, Fisher, 1 mg/ml) was applied and the ganglia were mixed to allow for further dissociation with a polished Pasteur pipette. The tissue was then filtered through 70 μm nylon cell strainer (Falcon) and re-suspended in DMEM F-12 GlutaMax media (Invitrogen) that contained 10% fetal bovine serum (FBS, Hyclone) and 1x penicillin streptomycin (Pen-Strep). The media also contained NGF (10 ng/ml, Millipore) and cytosine arabinoside (Ara-C, 2.4ug/ml, Sigma) to reduce proliferation of glia and fibroblasts. Neurons were cultured for seven days on 12 well plates coated with poly-D-Lysine (Falcon) for Western blot experiments or on 12 mm glass coverslips (#1 thickness, Chemglass) in a 24-well tissue culture plate (Falcon) at 37° C with 95% air and 5% CO2. On day 4, Ara-c was removed from the media and excluded for the remaining 3 days. On the day of the experiment, drugs were diluted into DMEM F-12 plus GlutaMax media and added directly onto the neurons at a 10X concentration without any wash.

*Western Blotting:*

Harvested tissues were homogenized in lysis buffer containing 10mM HEPES at pH 7.9, 20mM NaCl, 1mM EDTA, 0.05% Triton X-100, protease inhibitor cocktail (P8340, Sigma) and phosphatase inhibitor cocktail 2/3 (P5726, P0044, Sigma). Then the lysate was spun at 1000 x g for 15 minutes 4°C and the supernatant was collected and protein concentration determined using BCA Protein Assay Kit (PI-23223, Thermo Scientific, Waltham, MA). Twenty µg of protein per sample was mixed with loading buffer, 4X Laemmli Sample Buffer (Biorad, Hercules, CA, USA), with 5% dithiothreitol (Biorad) and heated to 95° C for 5 min to facilitate denaturation of protein and then ran on 7.5% sodium dodecyl sulfate polyacrylamide gel electrophoresis for separation. When the run was completed, the gels were transferred onto PVDF membranes (Millipore) overnight at 4°C. The following day the membranes were blocked with 5% dry milk for 1 hr at room temperature and then incubated with primary antibody overnight at 4° C. These membranes were then incubated in the horseradish peroxidase-conjugated secondary antibody (Jackson Immunoresearch, West Grove, PA) for chemiluminescent detection onto a radiography film or on a ChemiDoc Touch machine (Biorad) after the substrate (WBKLS0500, Millipore) was applied to the membranes. Densitometric analyses were performed on immunoblots with Image J software (NIH, Bethesda, MD) using the gel analysis tool available as a plugin from McMaster University (www.macbiophotonics.ca). Densitometry was done following instructions given for this plugin for ImageJ. Antibodies used are shown in Table 1.

*Immunocytochemistry (ICC):*

Seven days after establishment of DRG cultures, treatments were done to assess ACC phosphorylation, P body formation [1] and protein puromycelation. Cells were treated with drug concentrations applied from 10X stocks in DMEM F-12 plus GlutaMax, including FBS and Pen-Strep. Following treatment, cells were fixed in ice cold 10% formalin in phosphate buffered saline (PBS) for 1 hr. Cells were then washed with PBS and permeabilized in PBS containing 10% heat inactivated normal goat serum (NGS, Atlanta Biologicals, Atlanta, GA) and 0.02% Triton X-100 (Sigma) in PBS for 30 min and then blocked in 10% NGS in PBS for at least 1 hr. Following additional washes, primary antibodies were applied overnight at 4° C and the next day appropriate secondary antibodies (goat anti rabbit – alexafluor 488 or goat anti mouse – alexafluor 565, Invitrogen) were applied for 1 hour. After additional PBS washes, coverslips were mounted on frosted slides with ProLong Gold antifade (Invitrogen). For the puromycelation, surface sensing of translation (SUnSET) assay [51], puromycin (1 μM, Sigma) was applied during the last 15 min of the 1 hr incubation with drug. Immediately following the puromycin incubation, cells were washed in chilled HBSS containing 0.00036 % digitonin (Sigma) for 2 min prior to fixation for removal of background puromycin.

*Image Analysis:*

Images were taken on an Olympus Fluoview FV1200 laser scanning confocal microscope and analyzed using the colocalization tool within Olympus’ FV software. The intensity of each channel was adjusted so that only areas that contained a strong signal of both 488nm and 568nm were visible. This adjusted imaged contained distinct puncta that could then be counted and analyzed using Graphpad prism 6.

*R419 Specificity testing:*

Assays for inhibition of human cytochrome p450 enzymes or interference with ligand binding at an array of receptors shown in supplementary table 1 were all done in the presence of 10 μM R419, a concentration of drug at least 30 times higher than the concentration needed to stimulate AMPK activity in cells. All assays were performed at Eurofins Panlabs Taiwan, Inc. Representative references for the enzyme and ligand binding assays are: [10; 59].

*Electrophysiology:*

Mice were anesthetized with isoflurane (Vedco Inc., St. Joseph, MO) and sacrificed by decapitation. DRGs were dissected and placed in ice-cold HBSS (divalent free). Ganglia were incubated for 25 min in 20 U/ml Papain (Worthington, Lakewood, NJ) followed by 25 min in 3 mg/ml Collagenase Type II (Worthington). After trituration through a fire-polished Pasteur pipette, cells were plated on poly-D-lysine and laminin (Sigma) coated plates. Cells were allowed to adhere for several hours at room temperature in a humidified chamber and then nourished with Liebovitz L-15 medium (Life Technologies, Grand Island, NY) supplemented with 10% FBS, 10 mM glucose, 10 mM HEPES and 50 U/ml penicillin/streptomycin. To access NGF-induced excitability, DRG neurons were treated with 50 ng/ml NGF (Millipore). Neurons were used within 24 hr post plating.

Whole cell patch-clamp experiments were performed on isolated DRG neurons *in vitro* using a MultiClamp 700B (Axon Instruments) patch-clamp amplifier and PClamp 9 acquisition software (Axon Instruments). Recordings were sampled at 20 kHz and filtered at 1 kHz (Digidata 1322A, Axon Instruments). Pipettes (OD: 1.5mm, ID: 0.86mm, Sutter Instrument) were pulled using a P-97 puller (Sutter Instrument) and heat polished to 1.5-4 MΩ resistance using a microforge (MF-83, Narishige). Series resistance was typically < 7 MΩ and was compensated 60-80%. Data were analyzed using Clampfit 10 (Molecular Devices) and Origin 8 (OriginLab). Action potentials were elicited by injecting slow ramp currents from 0.1 to 0.7 nA with Δ = 0.2 nA over 1 sec to mimic slow depolarization. The pipette solution contained (in mM) 140 KCl, 11 EGTA, 2 MgCl_2_, 10 NaCl, 10 HEPES, 1 CaCl_2_pH 7.3 (adjusted with N-methyl glucamine), and was ~ 320 mosM. External solution contained (in mM) 135 NaCl, 2 CaCl_2_, 1 MgCl_2_, 5 KCl, 10 Glucose, 10 HEPES, pH 7.4 (adjusted with N-methyl glucamine), and was ~ 320 mosM. The osmolarity of each solution was adjusted with sucrose solution. Small diameter DRG neurons (15 – 22 pF) with an average size of 18 μm were used for all experiments. All recording were done at room temperature. For current clamp recordings, the seal stability and resting membrane potential (RMP) were examined and neurons with an initial seal resistance of < 5 GΩ, membrane blebs or high leak currents (> 0.2 nA) or with RMP more positive than -45mV were excluded.

*R419 Measurements*

R419 was injected into the hindpaws of naïve Swiss Webster mice at a dose of 10 μg in 25 μL sterile saline and hindpaw tissue was taken from terminally anesthetized mice at the indicated time points. Tissue was then placed in lysis buffer without phosphatase and protease inhibitor cocktails. Tissue was homogenized using a Precellys Minilys (Bertin Technologies) machine with a hard tissue grinding kit (MK28) according to the manufacture specifications. Homogenized tissue was then removed from the homogenizing beads and placed in a fresh sample tube and frozen at -80° C until analysis of R419 concentrations by liquid chromatography mass spectrometry (LCMS).

*Data Analysis and Statistics*

All data are presented as mean ± SEM. Data were analyzed with GraphPad (San Diego, CA, USA) Prism Version 6 for PC or Mac. Statistical tests used are described in figure legends. The *a priori* level of significance was set at 95%.

**Supplemental Table 1:**

| Receptor, enzyme or ligand | Species | % inhibition at 10µM |
| --- | --- | --- |
| CYP450, 1A2 | human | 0 |
| CYP450, 2C19 | human | 11 |
| CYP450, 2C9 | human | 6 |
| CYP450, 2D6 | human | 1 |
| CYP450, 3A4 | human | 12 |
| Adenosine A1 | human | -5 |
| Adenosine A2A | human | -5 |
| Adenosine A3 | human | -2 |
| Adrenergic α1A | rat | 29 |
| Adrenergic α1B | rat | 41 |
| Adrenergic α1D | human | 10 |
| Adrenergic α2A | human | 12 |
| Adrenergic β1 | human | 9 |
| Adrenergic β1 | human | 9 |
| Androgen (Testosterone), Androgen receptor | rat | -4 |
| Bradykinin B1 | human | 9 |
| Bradykinin B2 | human | 2 |
| Calcium Channel L-Type, Benzothiazepine | rat | 18 |
| Calcium Channel L-Type, Dihydropyridine | rat | 31 |
| Calcium Channel N-Type | rat | 1 |
| Cannabinoid CB1 | human | -9 |
| Dopamine D1 | human | -2 |
| Dopamine D2s | human | 1 |
| Dopamine D3 | human | 6 |
| Dopamine 4.2 | human | -8 |
| Endothelin ET_A | human | -1 |
| Endothelin ET_B | human | 6 |
| Epidermal Growth Factor (EGF) | human | 1 |
| GABA_A, Flunitrazepam, Central | rat | -1 |
| GABA_A, Muscimol, Central | rat | 12 |
| GABA_B1A | human | 1 |
| Glucocorticoid | human | -8 |
| Glutamate, Kainate | rat | 2 |
| Glutamate, NMDA, Agonism | rat | 28 |
| Glutamate, NMDA, Glycine | rat | -13 |
| Glutamate, NMDA,Phencyclidine | rat | 4 |
| Histamine H1 | human | 15 |
| Histamine H2 | human | 12 |
| Histamine H3 | human | 33 |
| Imidazoline I2, Central | rat | 5 |
| Interleukin IL-1 | mouse | -8 |
| Leukotriene, Cysteinyl CysLT1 | human | -3 |
| Melatonin MT1 | human | -3 |
| Muscarinic M1 | human | 7 |
| Muscarinic M2 | human | 5 |
| Muscarinic M3 | human | -7 |
| Neuropeptide Y Y1 | human | -1 |
| Neuropeptide Y Y2 | human | 11 |
| Nicotinic Acetylcholine | human | 8 |
| Nicotinic Acetylcholine α1,Bungarotoxin | human | -1 |
| Opiate δ (OP1, DOP) | human | -1 |
| Opiate κ (OP2, KOP) | human | 24 |
| Opiate μ (OP3, MOP) | human | 7 |
| Phorbol Ester | mouse | 0 |
| Platelet Activating Factor (PAF) | human | 26 |
| Potassium Channel [K_ATP] | hamster | 4 |
| Potassium Channel hERG | human | 35 |
| Prostanoid EP4 | human | -7 |
| Purinergic P2χ | rabbit | 15 |
| Purinergic P2γ | rat | 4 |
| Rolipram | rat | 11 |
| Serotonin (5-Hydroxytryptamine) 5-HT1A | human | -3 |
| Serotonin (5-Hydroxytryptamine) 5-HT2B | human | 9 |
| Serotonin (5-Hydroxytryptamine) 5-HT3 | human | 3 |
| Sigma σ1 | human | 42 |
| Sodium Channel, Site 2 | rat | 5 |
| Tachykinin NK1 | human | 13 |
| Thyroid Hormone | rat | 4 |
| Transporter, Dopamine (DAT) | human | 59 |
| Transporter, GABA | rat | 6 |
| Transporter, Norepinephrine (NET) | human | 7 |
| Transporter, Serotonin (5-Hydroxytryptamine) (SERT) | human | 18 |

**Off-target activity of R419**

Biochemical assay results are presented as the percent inhibition of specific binding to the targets or activity of the enzymes in the presence of 10 µM R419. All assays were performed at Eurofins Panlabs Taiwan, Inc.
